# P09-07 Physical activity and sedentary behaviour patterns among French adults during the COVID-19 health crisis

**DOI:** 10.1093/eurpub/ckac095.137

**Published:** 2022-08-29

**Authors:** Florian Manneville, Laurent Muller, Abdou Yacoubou Omorou

**Affiliations:** 1 APEMAC, Université de Lorraine, Vandoeuvre-Lès-Nancy, France; 2 CIC-1433 Clinical Epidemiology, CHRU de Nancy, Vand?uvre-lès-Nancy, France; 3 APEMAC, Université de Lorraine, Metz, France

**Keywords:** Physical activity, Sedentary Behaviour, Adults, COVID-19 health crisis

## Abstract

**Background:**

The COVID-19 health crisis and the various restrictions (lockdowns) implemented may have impacted individuals' behaviours (e.g. physical activity [PA] and sedentary behaviour [SB]) and psychological health (e.g., self-esteem or adjustment strategies to cope with stressful events). The objective of this study was to identify PA and SB patterns and to investigate their associations with socioeconomic and psychological characteristics among French adults during the COVID-19 health crisis.

**Methods:**

Cross-sectional data of French adults were collected during the COVID19 health crisis (between March 2020 and February 2021). PA and SB were measured using the International Physical Activity Questionnaire. The Rosenberg Self-Esteem Scale and the Brief Cope questionnaire were used to measure self-esteem and coping strategies, respectively. PA and SB cross-sectional patterns were identified using latent class analysis. Multivariable logistic regression models were used to investigate associations between identified patterns and adults' socioeconomic factors, self-esteem, and coping strategies.

**Results:**

Among the 241 included adults (mean age ± standard deviation: 29.6 ± 13.1 years), three cross-sectional PA and SB patterns were identified:

- sedentary walker (n = 141; 58.5%);
- varied PA practitioner (n = 68; 28.2%);
- walker with intense PA (n = 32; 13.3%);

Compared to the sedentary walker pattern, the walker with intense PA one was overrepresented by socially less advantaged adults, using more planning and less religion as coping strategies to stressful events, and those in the varied PA practitioner pattern used more denial as coping strategy.

**Conclusions:**

More than half of adults were in the least healthy pattern (sedentary walker). These results suggest using PA and SB as levers to cope with stressful life events.

